# Simultaneous Presence of Follicular Lymphoma, Diffuse Large B-cell Lymphoma, and Hodgkin-like Lymphoma

**DOI:** 10.4274/tjh.2018.0183

**Published:** 2018-11-13

**Authors:** Alexandra Papoudou-Bai, Leonidas Marinos, Konstantina Papathanasiou, Panagiotis Kanavaros, Eleni Kapsali

**Affiliations:** 1 University of Ioannina Faculty of Medicine, Department of Pathology, Ioannina, Greece; 2 Evangelismos General Hospital, Clinic of Hematopathology, Athens, Greece; 3 University of Ioannina Faculty of Medicine, Department of Anatomy-Histology-Embryology, Ioannina, Greece; 4 University of Ioannina Faculty of Medicine, Department of Hematology, Ioannina, Greece

**Keywords:** Transformation, Follicular lymphoma, Hodgkin-like lymphoma

## To the Editor,

**Figure 1:**
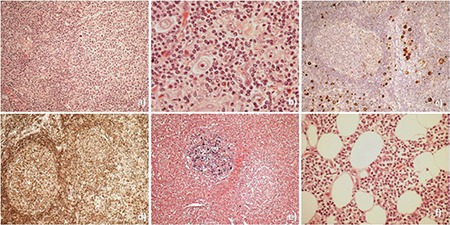
a) Synchronous presence of follicular lymphoma (FL) with Hodgkin-like lymphoma (hematoxylin and eosin staining, 200^x^); b) Hodgkin cells admixed with scattered eosinophils, plasma cells, and histiocytes (hematoxylin and eosin staining, 600^x^); c) CD30+ Hodgkin and Reed-Sternberg cells in the extrafollicular areas surrounding a neoplastic follicle (immunohistochemical staining, 100^x^); d) synchronous presence of FL with Hodgkin-like lymphoma, where the neoplastic follicles express the BCL2 protein (immunohistochemical staining, 100^x^); e) Epstein-Barr virus (EBER)-positive cells in the neoplastic follicles (in situ hybridization, 100^x^); f) diffuse large B-cell lymphoma (hematoxylin and eosin staining, 400^x^).

Follicular lymphoma (FL) is a relatively indolent B-cell lymphoma that may transform to a higher-grade lymphoma, most commonly diffuse large B-cell lymphoma (DLBCL) [1]. On the other hand, the occurrence of Hodgkin lymphoma (HL) subsequent to FL as well as composite lymphomas that are composed of HL and FL have rarely been recorded [1,2,3,4,5]. To the best of our knowledge, this is the first reported case of the simultaneous presence of FL, DLBCL, and Hodgkin-like lymphoma in a lymph node. A 65-year-old man developed a palpable mass in his left axilla, which grew larger in a period of 2 months. The patient reported no other symptoms and had no notable medical history. Biopsy of the left axillary lymph node revealed grade 3A-FL with areas of DLBCL. DLBCL was also observed in the biopsy of a mass of the thoracic wall, which was near the enlarged axillary lymph node. Moreover, in the lymph node, Hodgkin and Reed-Sternberg (HRS) cells were identified in extrafollicular areas and some neoplastic follicles (Figure 1). Although some scattered eosinophils, plasma cells, and histiocytes were observed in the cellular background of the extrafollicular HRS cells, the extent of this cellular infiltrate was less than what would be expected for typical HL (Figure 1). Immunohistochemistry revealed that the follicular neoplastic cells were CD20+, CD10+, BCL6+, BCL2+, PAX-5+, CD30-, CD15-, and MUM1- (Figure 1). The HRS cells were CD30+, CD15+ (20%), CD20-, CD10-, BCL6-, MUM1+, CD3-, CD4-, CD8-, and weakly PAX-5+ (Figure 1). A few reactive follicles with CD10+, BCL6+, and BCL2- germinal center cells were also observed. EBER-in situ hybridization demonstrated Epstein-Barr virus (EBV) positivity in some cells in a few neoplastic follicles (Figure 1), but not in the DLBCL component or in the HRS cells. The above findings were consistent with the simultaneous presence of FL, DLBCL, and Hodgkin-like lymphoma. Computed tomography (CT) and positron-emission tomography (PET)/CT and bone marrow (BM) biopsy were performed. The lymphoma was assigned stage IV because of BM infiltration. The BM lymphoid infiltration was diffuse (15%-20% of the total BM nucleated cells) and composed of medium-sized lymphoid cells with immunophenotype of CD20+, CD10+, BCL6+, PAX-5+, MUM1-, and CD30-. In addition, some cells with the morphology of Hodgkin cells and immunophenotype of CD30+, CD15+, CD45+, CD20-, CD10-, BCL6-, and PAX-5- were also identified in the lymphoid infiltration. The patient subsequently underwent six cycles of rituximab-CHOP chemotherapy without adverse effects. After treatment, the CT scans and PET/CT results were consistent with complete response and BM biopsy showed no lymphoma. He is currently in regular follow-up. In our case, the DLBCL component may correspond to transformation of the FL component, and the EBV-negative Hodgkin-like component may arise from the EBV-negative intrafollicular HRS cells that we detected in the lymph node. The occurrence of HL subsequent to FL as well as composite lymphomas consisting of HL (with classical immunophenotype) and FL without EBV association were rarely reported [2,3,4]. In contrast, Menon et al. [5] described transformation of FL to EBV-positive Hodgkin-like lymphoma. Interestingly, in keeping with the findings of Menon et al. [5], we also observed EBV-positive cells in a few neoplastic follicles. This suggests that EBV infected the cells secondarily in the neoplastic follicles. In conclusion, this is the first reported case of the simultaneous presence of FL, DLBCL, and EBV-negative Hodgkin-like lymphoma.

## References

[1] Fischer T, Zing NPC, Chiattone CS, Federico M, Luminari S (2018). Transformed follicular lymphoma. Ann Hematol.

[2] Brauninger A, Hansmann ML, Strickler JG, Dummer R, Burg G, Rajewsky K, Küppers R (1999). Identiﬁcation of common germinal-center B-cell precursors in two patients with both Hodgkin’s disease and non-Hodgkin’s lymphoma. N Engl J Med.

[3] Marafioti T, Hummel M, Anagnostopoulos I, Foss HD, Huhn D, Stein H (1999). Classical Hodgkin’s disease and follicular lymphoma originating from the same germinal center B cell. J Clin Oncol.

[4] Nakamura N, Ohshima K, Abe M, Osamura Y (2007). Demonstration of chimeric DNA of bcl-2 and immunoglobulin heavy chain in follicular lymphoma and subsequent Hodgkin lymphoma from the same patient. J Clin Exp Hematop.

[5] Menon MP, Hutchinson L, Garver J, Jaffe ES, Woda BA (2013). Transformation of follicular lymphoma to Epstein-Barr virus-related Hodgkin-like lymphoma. J Clin Oncol.

